# Microfluidic Platform for the Elastic Characterization of Mouse Submandibular Glands by Atomic Force Microscopy

**DOI:** 10.3390/bios4010018

**Published:** 2014-02-27

**Authors:** Aaron P. Mosier, Sarah B. Peters, Melinda Larsen, Nathaniel C. Cady

**Affiliations:** 1 State University of New York (SUNY) College of Nanoscale Science & Engineering, 257 Fuller Rd., Albany, NY 12203, USA; E-Mail: amosier@albany.edu; 2 Department of Biological Sciences, State University of New York at Albany, 1400 Washington Avenue, Albany, NY 12222, USA; E-Mails: speters2@albany.edu (S.B.P.); mlarsen@albany.edu (M.L.)

**Keywords:** microfluidics, elasticity, tissue engineering, submandibular gland, AFM

## Abstract

The ability to characterize the microscale mechanical properties of biological materials has the potential for great utility in the field of tissue engineering. The development and morphogenesis of mammalian tissues are known to be guided in part by mechanical stimuli received from the local environment, and tissues frequently develop to match the physical characteristics (*i.e*., elasticity) of their environment. Quantification of these material properties at the microscale may provide valuable information to guide researchers. Presented here is a microfluidic platform for the non-destructive *ex vivo* microscale mechanical characterization of mammalian tissue samples by atomic force microscopy (AFM). The device was designed to physically hold a tissue sample in a dynamically controllable fluid environment while allowing access by an AFM probe operating in force spectroscopy mode to perform mechanical testing. Results of measurements performed on mouse submandibular gland samples demonstrate the ability of the analysis platform to quantify sample elasticity at the microscale, and observe chemically-induced changes in elasticity.

## 1. Introduction

Xerostomia, commonly known as “dry-mouth”, is the subjective feeling of oral dryness that is commonly associated with hyposalivation [1]. It is a diagnosed medical condition frequently presented as the unintended side effect of certain medications or radiation therapies. It is also a symptom of an autoimmune disease known as Sjögren’s syndrome, while in other cases the exact cause is unknown [2]. Xerostomia is most commonly due to a deficiency of saliva production in the mouth, which may, in turn, lead to additional medical complications, such as dental caries and halitosis due to a shift in oral microbiota. In severe cases, patients have difficulty chewing and swallowing food.

The largest salivary glands present in mammals are the parotid, the submandibular (SMG), and the sublingual glands. They exist in pairs and are responsible for the majority of saliva production. Structurally each gland is composed of a series of acinar “buds”, which produce saliva and are connected by a series of ducts that guide the saliva into the oral cavity. Medications, head or neck radiation, and Sjögren’s syndrome can destroy acinar cells, reducing or destroying an individual's ability to produce saliva. Research efforts are therefore being directed at developing engineered salivary tissue that could be transplanted into a patient to restore normal salivation capability.

*In vitro* studies using mice as a model organism have shown that the mechanical properties of the local environment play a large role in guiding the proper morphological and functional development of embryonic salivary glands, particularly substrate elasticity [3,4]. To achieve the goal of engineering functional salivary glands, it is necessary to quantify the mechanical characteristics of the engineered substrates as well as those of the developing and adult salivary tissues. Several analytic techniques exist to perform such measurements, one of the most recently developed being nanoindentation, which has become an accepted method for determining sample elasticity (among other properties) and is especially useful for determining the micro-scale mechanical behavior of samples. Atomic force microscopy [5], operated in force spectroscopy mode, is able to make nanoindentation measurements of material samples and has been employed in recent years to perform these measurements on a variety of soft samples including biological materials [6]. AFM offers several advantages over more traditional techniques for mechanical analysis (such as rheometry) including microscale spatial resolution, minimally-destructive repeatable measurement, and the ability to couple with optical analysis.

Presented here are the results of mechanical measurements of living salivary gland tissue excised from adult and embryonic mice. These measurements were performed using a novel microfluidic device capable of holding a tissue sample in place under controlled fluid flow and, when placed on the stage of an atomic force microscope, allows for mechanical testing of that sample by AFM force spectroscopy. Elastic moduli of adult pregnant mouse glands were measured under static fluid conditions, as well as that of glands taken from 13 day old embryos. These data illustrate the ability of the developed micro-device, when coupled with an atomic force microscope (AFM), to quantify the elasticity of relatively large samples in an *ex situ* manner. This work was then extended to show that a dynamic shift in sample elasticity could be chemically induced and recorded. To our knowledge, there are no reports in the literature of similar elasticity measurements being performed on mouse SMGs using any technique, with the exception of Peters, *et al*., to which the authors of this work contributed [3].

The cytoskeletal structure of epithelial and mesenchymal cells within the salivary glands is, in part, comprised of actin filaments which dock at the cell membrane and cross the interior of the cell. Tension along actin filaments counterbalances the osmotic pressure of the cytoplasm to help give rise to cell morphology and mechanical characteristics. The integrity of the actin cytoskeleton directly impacts elasticity of the cell [7]. The small molecule, blebbistatin, has been found to play a role in disrupting the interaction of actin with non-muscle myosin type II, which together control cellular contractility [8,9]. Additionally, previous work indicates that blebbistatin interrupts the necessary intracellular contractility involved in mSMG branching morphogenesis [10]. The activity of blebbistatin is most pronounced at physiological temperatures, and greatly reduced or nonexistent at room temperature. Based on blebbistatin’s known effect of disrupting actomyosin contractility and branching morphogenesis in the mSMG, this drug was chosen as a representative chemical agent to elicit and record rapid shifts in elastic modulus of an SMG sample using the microfluidic device. As the elasticity of an engineered tissue often correlates with its desired functionality, this device demonstrates its utility as a tool to aid in the field of tissue engineering, providing researchers a means of characterizing samples developed *in vitro*, and benchmarking against *ex vivo* tissue*.*

## 2. Experimental Section

### 2.1. Microfluidic Device

#### 2.1.1. Description and Function

**Figure 1:**
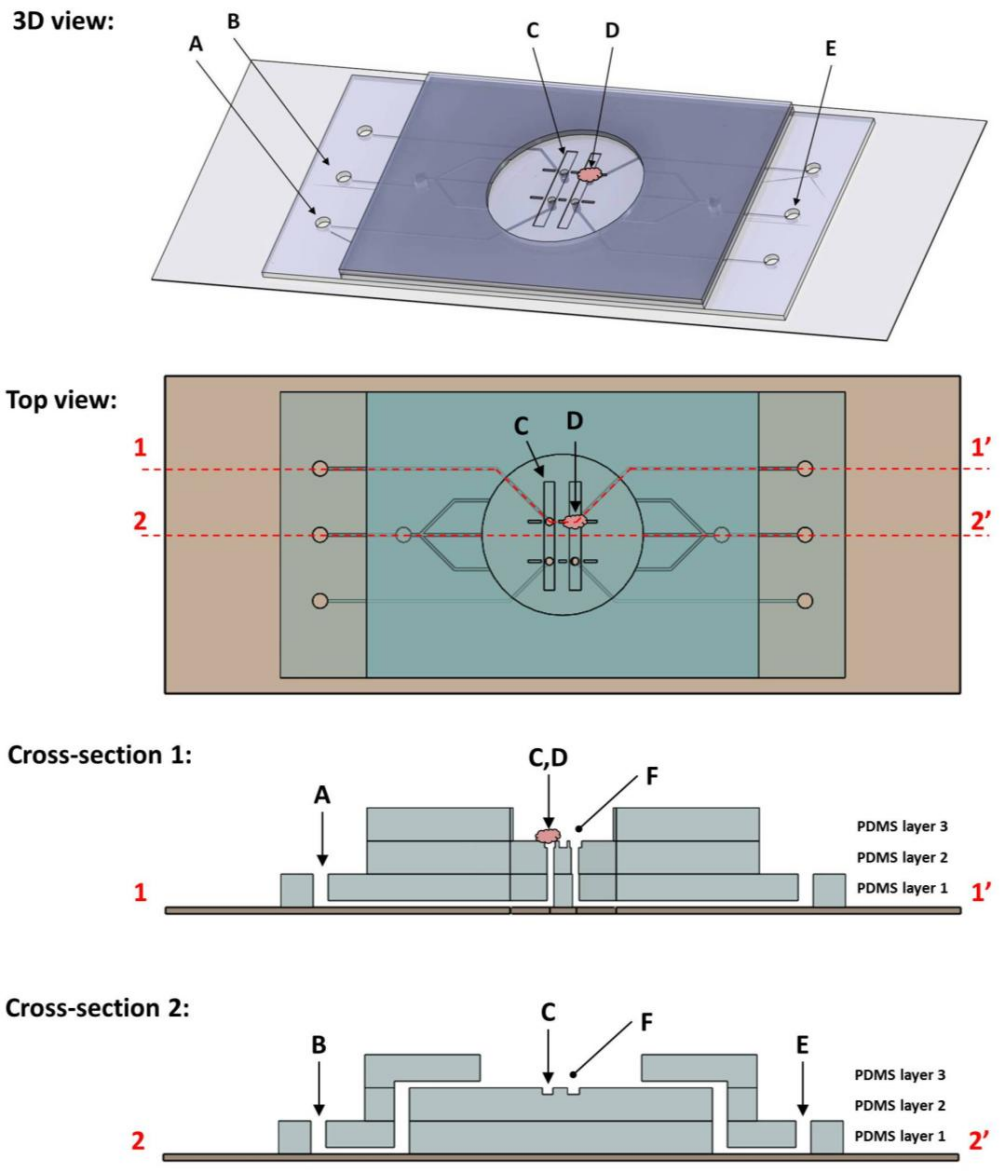
Shown here are schematic diagrams of the microfluidic clamp device, including (from top to bottom) a three dimensional model, top-down planar view, and cross-sectional slice views. The device itself measures 43 mm by 22 mm and is assembled on a standard 75 × 25 mm glass microscope slide and three layers of patterned polydimethylsiloxane (PDMS). Cross sections are taken along the dotted lines indicated in the top view. Note that the cross-sections have been stretched in the Z-direction for clarity and are not to scale. Features of note include four fluid chucks (a tissue sample immobilized by one chuck (**D**) and syringe connection port (**A**) are labeled here), stabilization trenches (**C**) patterned within the floor of the analysis chamber (**F**), as well as fluid perfusion channels (inlet (**B**) and outlet (**E**)).

The microfluidic flowcell device (Figure 1) measured 4.3 cm × 2.2 cm × 3.1 mm. It consisted of three layers of patterned polydimethylsiloxane (PDMS) bonded to the surface of a glass microscope slide. Layers 1 and 2 were cast to measure approximately 0.8 mm thick, and layer three approximately 1.5 mm thick. At the center was an analysis region where the tissue samples were held in place by action of a fluid “chuck” built into the device. The chuck was an opening present in the analysis region in which negative fluid pressure was applied via a syringe connected by embedded channels. The pressure differential between the chuck channel and analysis region acted to hold the sample to that surface. Features patterned on the bottom PDMS layer formed the fluid chuck channels. A second layer created the floor of the analysis region, which patterned with trenches. At the center of the trenches were the chuck holes, vias cut to the layer 1 channels. The top PDMS layer defined the edge of the analysis region and acted as a fluid reservoir to maintain sample hydration. It also contained fluid channels designed to perfuse liquid across the sample during measurement.

The device was designed to be integrated into a combined atomic force microscope and confocal laser scanning microscope (AFM/CLSM). Four fluid chucks were present in the floor of the analysis region, each measuring 100 µm in diameter and connected to peripheral tubing ports via channels in the bottom PDMS layer. Tubing was connected to these ports and negative fluid pressure applied via syringe. Perfusion channels present were employed to create fluid flow across the analysis chamber. This served to sweep away any debris or loose cells present, but also to alter the chemistry of the local environment through the introduction of blebbistatin. Also present within the analysis chamber were trenches measuring 8.3 mm by 1 mm and 0.1 mm deep. The fluid chucks were in the floor of these trenches and they served to add lateral stability to samples being held by the chuck.

#### 2.1.2. Fabrication

Microfluidic devices were fabricated in batches and used only once each for a single experiment. Briefly, SU-8 was photolithographically patterned on Si wafers to form molds in which to cast the three PDMS layers using the method and materials described elsewhere [11]. PDMS was mixed at the standard 10:1 ratio, degassed, poured into the mold, and then baked at 60 °C for 90 min. The PDMS was then cut from the mold and diced into individual device components. Tubing holes and through vias were punched prior to device assembly. Layers were bonded one at a time using an air plasma treatment, starting with the base layer onto the glass, then adding the other layers in succession. A final 60 °C bake for 10 min was performed to cure the bonds, and then tubing was connected and glued.

### 2.2. SMG Sample Preparation

Mouse SMG organ explants were dissected from adults or from embryos removed from timed-pregnant females (strain CD-1, Charles River Laboratories) with the day of plug discovery designated as E0, following protocols approved by the University at Albany IACUC committee. Embryonic day 13 (E13, 4-5 buds), SMGs were microdissected from mandible slices and cultured as previously described [12]. Briefly, mandible slices were removed from embryos with a sterile scalpel and, from these, SMGs were microdissected using sterile forceps under a stereo dissecting microscope (SMZ645, Nikon). Dissected glands were measured within 4 h of removal from the organism and stored immersed in 1:1 DMEM/Ham’s F12 Medium (F12) lacking phenol red (Invitrogen) at 37 °C under 5% CO_2_ atmosphere. The medium was supplemented with 50 µg/mL transferrin, 150 µg/mL L-ascorbic acid, 100 µg/mL penicillin, and 100 µg/mL streptomycin, to make complete DMEM/F12 medium, as described previously [10].

### 2.3. Mechanical Analysis

#### 2.3.1. Measurement Procedure

Prior to measurement, the device reservoir was filled with DMEM/F12 media, and a small (300–600 µm diameter) piece of SMG explant was placed over the chuck hole in the analysis region of the device. Samples were roughly spherical or ovoid in shape, with a thickness always >100 µm. The sample was manipulated with forceps to hold it over the hole while manually withdrawing the plunger of a syringe connected to the chuck. Effort was made during measurement to gently maintain the minimum amount of negative chuck pressure required to prevent the sample from shifting. This required pressure varied from sample to sample. The AFM scan head was then immersed in the fluid and force spectroscopy performed.

Static fluid measurements were performed at room temperature. The device and sample were kept at 37 °C using heated stage insert with a Lake Shore 331 temperature controller (Lake Shore Cryotronics, Westerville, OH, USA). For experiments including fluid perfusion, the pharmacological inhibitor blebbistatin (Calbiochem, Cat#203391) was resuspended in DMSO vehicle and diluted in complete media to a concentration of 100 µM. The solution was then pumped through the device using a KD Scientific syringe pump at a flow rate of 50 µL/min; a rate which ensured complete fluid exchange within the analysis region in approximately 5 min.

#### 2.3.2. AFM Force Spectroscopy

AFM nanoindentation was performed on a Bruker BioScope Catalyst AFM (Bruker) mounted on a Leica SP5 confocal microscope (Leica Microsystems). Nanoindentation experiments were performed in closed-loop mode with probes consisting of silicon cantilevers of nominal spring constant 0.01 N/m, functionalized with 12 µm diameter borosilicate spheres. The actual spring constants of probes used were determined through use of the thermal tune method [13] to be 0.0158 N/m ± 5 × 10^−5^ and 0.0099 N/m ± 8 × 10^−5^. Ramp sizes of 3–10 µm with approach speeds of 5–8 µm/s were used. Force curve data were analyzed to produce modulus values using the Bruker Nanoscope Analysis software V1.4. Force curves from each measured spot were processed in separate batches. A boxcar filter was used to smooth the curve, applying a moving average over n = 5 data points. Next, baseline correction was performed. Finally, the Hertz model was fit to the approach portion data using a linearized model. A Poisson ratio of 0.5 was assumed.

## 3. Results and Discussion

### 3.1. Elastic Modulus of Adult and Embryonic SMGs in Media

**Figure 2:**
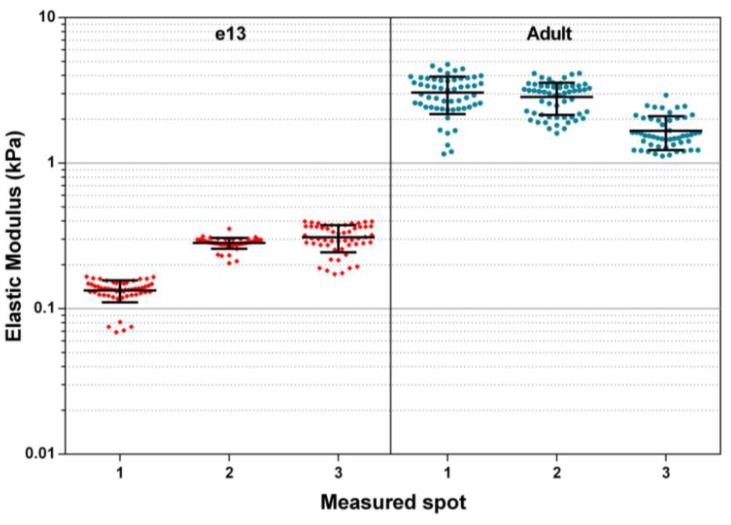
Elastic modulus of adult and e13 mouse submandibular glands (SMG) under static fluid conditions. Shown plotted are measured elastic modulus values of several spots on adult and embryonic SMG samples. Individual data points represent modulus values calculated from a single AFM force curve. Bars represent mean and standard deviations of values recorded at that particular sample spot.

Reported here are elastic moduli recoded from randomly located spot measurements performed on both adult SMG and embryonic day 13 (E13) samples (3 spots each). These data (Figure 2) represent a subset of measurements used to calculate average SMG moduli reported previously by Peters *et al.* [3]. Measured adult moduli ranged from 1.66 ± 0.44 kPa to 3.05 ± 0.88 kPa, and E13 moduli ranged from 0.13 ± 0.02 kPa to 0.31 ± 0.07 kPa. These measurements fall within the range previously reported of similar mammalian soft tissues measured by nanoindentation [14].

The variation deviation between moduli measured from spot to spot may be better understood by considering the complex structure of the sample being probed. As mentioned previously, submandibular glands are composed of several distinct tissue types, including the epithelium and encasing mesenchyme. Mesenchyme contains a fibrous network of ECM proteins, which are expected to confer a greater degree of structural rigidity than is present in epithelial cells. Within this mesenchyme are also a network of nerves and blood vessels. Furthermore, the epithelium is arranged physically into a three dimensional branched ductal structure terminating in end buds. At the interior of the buds and ducts in the adult gland are voids containing only the liquid media of the local environment. It is therefore logical to expect that elasticity measurements performed at different points on a sample are likely to yield varying results depending on the cell types or structures locally being probed.

It is unknown whether it is macroscale structural heterogeneity or microscale cell heterogeneity that accounts for the variation between spot measurements. However, the indentation depth used during measurements performed here was >1 µm, which is quite small compared to the size of the cells (5–20 µm in diameter). For this reason it is unlikely that subsurface heterogeneity (voids) contributed significantly to measurement deviation. More likely to influence spot to spot variation in data are heterogeneities existing on the size scale of the indenter, such as differing cell types and location on a particular cell being probed (*i.e*., center of cell body *vs*. cell-cell junction). Salivary epithelial cells that form the acinar units in adult tissues are known to be highly coordinated, forming tight junctions at their interfaces. These tight junctions are crucial to the directed secretion of saliva, and it is possible that a nanoindentation measurement centered on such a junction would yield a much higher elasticity than one made at the center of a cell body immediately adjacent to it. Despite variation between measured spots, these data, when taken as a whole, provide useful information to researchers seeking to understand the behavior of single cells in the context of a larger multi-cellular tissue system. 

### 3.2. Effect of Blebbistatin on SMG Elastic Modulus

**Figure 3:**
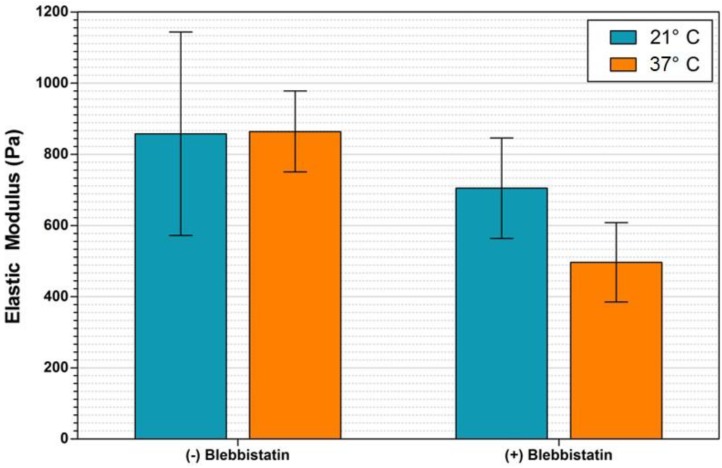
Elastic modulus of adult mouse submandibular glands (SMGs) under fluid flow conditions. Plotted here are elastic modulus data measured on SMG samples under fluid flow in the presence and absence of the small molecule inhibitor blebbistatin. Bar colors denote temperature. The addition of blebbistatin was observed to decrease the sample elasticity significantly for both temperatures, with the greatest effect detected at 37 °C.

Additional experimentation was performed using the microfluidic device to determine the elasticity of SMG samples under flow conditions, and to record an environmentally induced shift in that elasticity. The experiment was performed at two temperatures, 37 °C, corresponding to the physiological condition optimal for blebbistatin activity, and 21 °C, room temperature corresponding to non-ideal conditions to limit blebbistatin activity. Results of mouse SMG elastic modulus measurements performed under fluid flow are shown in Figure 3. Measurements taken immediately after removal from the CO_2_ incubator and prior to the addition of blebbistatin indicate equivalent sample moduli of 858 ± 286 Pa and 864 ± 113 Pa, at 21 °C and 37 °C, respectively. Measurements recorded over the course of several minutes one hour following the addition of 100 μM blebbistatin, during which time temperature was maintained, indicate the elasticity of the sample at 21 °C decreased approximately 18% to 705 ± 141 Pa, while the elasticity of the 37 °C sample decreased approximately 43% to 496 ± 112 Pa. 

Sample elasticity was nearly identical between the two temperature SMG samples prior to the application of the blebbistatin. Subsequent to the addition of the chemical inhibitor, the sample at room temperature decreased in modulus by 153 Pa (18%), while the sample at elevated temperature decreased in modulus by 367 Pa (38%). At both temperatures, the effect of blebbistatin on sample elastic modulus was statistically significant, as determined by 1-way ANOVA using an unpaired t-test with P < 0.05. These results confirm that blebbistatin is effective at decreasing the elastic modulus of adult mouse submandibular glands, and that the AFM nanoindentation coupled with the described microfluidic device is able to perceive and record this shift. Longer-term experimentation would be required to determine if gland elasticity would continue to decrease to a specific minimum value regardless of temperature.

## 4. Conclusions

Presented here is a microfluidic flowcell device for the non-destructive fixation of mammalian tissue samples, which when integrated into an AFM enables the microscale quantification of sample mechanical properties within a dynamically controllable fluid environment. Data presented relating to the elastic modulus of measured adult and embryonic mouse salivary glands demonstrates the utility of the platform for measuring the mechanical properties of soft biological tissue samples. It does so without the need for chemical surface fixation or other artificial means of sample immobilization that could potentially introduce additional unwanted experimental variables. This unique capability allows for the power of AFM nanoindentation to be brought to bear in a manner that minimally impacts the sample being interrogated, allowing for more accurate and relevant measurements to be performed. Furthermore, the system is able to perceive dynamic macroscale shifts in the mechanical properties of tissues over relatively short time periods.

Additionally, the scope of material properties quantifiable with AFM force spectroscopy is not limited to elasticity. Use of this device may also allow for investigation of sample viscous properties, creep behavior, and adhesion. The ability to perform such dynamic microscale-resolved biological material characterization may prove to be of great use in the field of tissue engineering, as it will allow for a greater level of detailed comparison between tissues developed in the laboratory and those present in live animals.
